# Antimicrobial Resistance Profiles of *Staphylococcus* spp. Isolated from the Oral Cavity of Captive Wild Mammals at a Zoo in Southern Brazil

**DOI:** 10.3390/ani16203283

**Published:** 2026-10-18

**Authors:** Francieli Gesleine Capote Bonato, Ana Cláudia Souza Andrade, Henrique Susumu Tanaka, Jorge Fernandes de Azevedo, Kariny Aparecida Jardim Rubio, Halison Murilo da Silva Oliveira, Heloiza de Oliveira Marchi, Vitor Yukio Takahashi, Bruna Pereira Bitencourt da Silva, Rodrigo Neca Ribeiro, Wesley Alves Secundini, Gabriela Catuzo Canonico Silva, Denis Vinicius Bonato, Ranulfo Piau Junior, Zilda Cristiani Gazim, Lidiane Nunes Barbosa, Daniela Dib Gonçalves

**Affiliations:** 1 Programa de Pós-Graduação em Ciência Animal com Ênfase em Produtos Bioativos, Universidade Paranaense, Umuarama 87502-210, Paraná, Brazil; 2 Curso de Medicina Veterinária, Universidade Paranaense, Umuarama 87502-210, Paraná, Brazil; 3 Zoológico Municipal, Curitiba 81860-270, Paraná, Brazil; 4 Mestrado Profissional de Plantas Medicinais e Fitoterápicos na Atenção Básica, Universidade Paranaense, Umuarama 87502-210, Paraná, Brazil; 5 Programa de Pós-Graduação em Biotecnologia Aplicada a Agricultura, Universidade Paranaense, Umuarama 87502-210, Paraná, Brazil

**Keywords:** wild mammals, captive animals, antimicrobial susceptibility, multidrug-resistant bacteria, One Health

## Abstract

Antimicrobial resistance in *Staphylococcus* spp. poses a challenge to One Health due to the potential for transmission among humans, animals, and the environment. This study investigated antimicrobial resistance patterns in *Staphylococcus* spp. isolates from the oral cavities of non-human primates and wild felids kept in captivity at a zoo in southern Brazil. Samples were collected from *Sapajus apella* and wild felids (*Leopardus pardalis*, *Leopardus guttulus*, *Herpailurus yagouaroundi*, *Leopardus wiedii*, *Panthera tigris*, *Puma concolor*, *Panthera onca*). Bacterial isolation and identification were performed using phenotypic methods, followed by antimicrobial susceptibility testing and molecular diagnosis for the detection of *Staphylococcus aureus* and the *mecA* gene. Of the *Staphylococcus* spp. isolates, 64.5% originated from non-human primates and 35.5% from wild felids. *S. aureus* was identified in 12.9% of the isolates, all from non-human primates. Azithromycin, erythromycin, and sulfamethoxazole-trimethoprim showed 90.3% resistance. Additionally, 80.6% of the isolates were multidrug-resistant (MDR), and 90.3% demonstrated a multiple antibiotic resistance (MAR) index, suggesting possible exposure to antimicrobial selective pressure. These findings demonstrate the circulation of multidrug-resistant *Staphylococcus* spp. in captive animals and reinforce the importance of antimicrobial resistance surveillance from a One Health perspective.

## Simple Summary

Antimicrobial resistance is a challenge to One Health. This study aimed to analyze the antimicrobial resistance profile of *Staphylococcus* spp. isolates from the oral cavities of non-human primates and wild felids kept in captivity at a zoo in southern Brazil. More than half of the *Staphylococcus* spp. isolates originated from non-human primates, and *S. aureus* was identified in only a small percentage (12.9%) of the isolates, all of which came from non-human primates. A large percentage of isolates exhibited multidrug resistance and elevated MAR indices, highlighting the occurrence of antimicrobial resistance among captive wild mammals. Thus, the data from this study suggest the circulation of multidrug-resistant *Staphylococcus* spp. among captive wild animals and demonstrate that monitoring antimicrobial resistance is of utmost importance for One Health.

## 1. Introduction

Antimicrobial resistance in *Staphylococcus* species is a global One Health concern, as this pathogen moves between the environment, animals, and humans [1]. Infections caused by these resistant pathogens are associated with therapeutic failure, increased morbidity, and public health impact [2,3,4]. The potential for the transmission of resistance genes across different interfaces requires intense surveillance and intervention [5].

Although bacteria of the genus *Staphylococcus* are frequently found colonizing the skin and mucous membranes of humans and various animal species [6,7], certain species, such as *Staphylococcus aureus*, have attracted medical interest due to their high virulence, ability to acquire antimicrobial resistance, and potential for transmission between humans and animals, a matter of relevance to animal production and global public health [2,4,6,7].

Wild animals have been described as potential reservoirs of *Staphylococcus aureus* capable of transmission to humans [8]. The presence of this pathogen in wild animals has been linked to increasing contact with human populations, facilitated by anthropogenic activities (indiscriminate use of antibiotics in medicine and livestock farming, and soil and water contamination) [9]. In captivity, this interaction is even more intense due to frequent contact with keepers, veterinarians, and visitors. Monitoring wild animal species is relevant as part of epidemiological surveillance [5].

Human interference in the wild environment poses a potential One Health risk [10,11]. This situation is relevant to wild species with significant ecological appeal. Non-human primates play an important biological role in forest regeneration by acting as seed dispersers, while wild felids perform fundamental functions within the ecosystem’s food web as predators, helping to maintain ecological balance [12,13]. However, due to anthropogenic activities, they can act as potential carriers of resistant bacteria, such as *Staphylococcus* spp., which are commonly found in human infections. These bacteria can adapt to different hosts and acquire resistance through mutations or horizontal gene transfer, favoring the emergence and dissemination of resistant phenotypes [4,8].

In this context, wild animals can act as sentinels, providing important information on potential public health risks due to the possibility of pathogen transmission, and given that antimicrobial resistance in mammals can vary according to taxonomy and trophic source [10,14].

Despite concerns regarding the transmission of multidrug-resistant microorganisms, studies on the oral colonization by *Staphylococcus* spp. and antimicrobial resistance in captive wild mammals, such as non-human primates and felids, in Brazilian zoos remain limited.

In this context, the surveillance of multidrug-resistant microorganisms in wild animals kept in zoos plays a strategic role, given that these environments foster a continuous interface among wildlife, humans, and the environment. Frequent exposure of animals to direct or indirect contact with visitors, keepers, and technical staff can facilitate the circulation and spread of resistant bacteria, reinforcing the need for continuous microbiological monitoring from a One Health perspective. Thus, the objective of this study was to investigate antimicrobial resistance patterns in *Staphylococcus* spp. isolates recovered from the oral cavity of non-human primates and wild felids kept in captivity at a municipal zoo in southern Brazil.

## 2. Materials and Methods

### 2.1. Ethical Aspects

The research was approved by the Chico Mendes Institute for Biodiversity Conservation (ICMBio) under protocol number 82499-1 and also by the Ethics Committee on Research Involving Animal Experimentation of the Paranaense University (UNIPAR) under protocol number 39559.

### 2.2. Sample and Collection Site

Sample collection was carried out at a municipal zoo in southern Brazil. The zoo animals included in the study originated from rescue and seizure operations carried out by environmental agencies, such as Water and Land Institute (IAT), Environmental Police, Brazilian Institute of the Environment and Renewable Natural Resources (IBAMA), and the Wildlife Support Center (CAFS). Only non-human primates and wild felids that had been at the zoo for more than a year were included in the study. Samples were collected from non-human primates (*Sapajus apella*) aged 5 to 23 years and wild felids, such as: *Leopardus pardalis* (aged 11 to 12 years), *Herpailurus yagouaroundi* (aged 5 to 9 years), *Leopardus guttulus* (aged 3 to 7 years), *Leopardus wiedii* (aged 10 to 11 years), *Panthera tigris* (aged 16 years), *Puma concolor* (aged 5 to 11 years), and *Panthera onca* (aged 10 to 12 years).

### 2.3. Animal Restraint

The capture and handling procedures for these animals were carried out by properly trained veterinarians and biologists from the zoo’s staff, in accordance with the specific species. The containment of wild felids and non-human primates was carried out using drugs, administered through dart shots using a blowgun, according to Andrade et al. [15].

### 2.4. Oral Sample Collection

Samples were collected during routine physical examinations using a sterile swab moistened with the transport medium; the swab was inserted into the animal’s oral cavity, pressed against the tissue with a rotating motion, then placed in the transport medium, kept refrigerated on ice, and immediately taken to the laboratory for processing.

### 2.5. Microbiological Diagnosis

The swabs were individually placed into tubes containing 3 mL of Brain Heart Infusion (BHI) medium and incubated at 37 °C for 24 h. After this period, the obtained cultures were streaked onto plates containing Mannitol Salt Agar and incubated at 37 °C for 24 h for the isolation of aerobic Gram-positive bacteria. Subsequently, each isolate was subjected to analysis of macroscopic characteristics, Gram staining, and biochemical tests [16]. The Gram-positive, catalase-positive cocci were subjected to the coagulase test for classification as coagulase-positive *Staphylococcus* or coagulase-negative *Staphylococcus*.

### 2.6. Conventional Antibacterial Susceptibility Tests

Antimicrobial susceptibility testing was performed using the agar disk diffusion method in accordance with the criteria established by the Clinical and Laboratory Standards Institute [17,18,19,20]. The antibiotics most used in both veterinary and human medical practice against staphylococcal infections served as the inclusion criteria for the antibiotics. Thus, the following antibiotics were tested: amikacin (30 μg), azithromycin (15 μg), ciprofloxacin (5 μg), cefepime (30 μg), cefoxitin (30 μg), erythromycin (15 μg), ertapenem (10 μg), gentamicin (10 μg), imipenem (10 μg), and trimethoprim-sulfamethoxazole (25 μg).

Previously isolated colonies were inoculated into BHI medium and incubated overnight to obtain bacterial growth for subsequent analyses. The bacterial inoculum was standardized according to the 0.5 McFarland scale, and the bacterial suspension was inoculated onto plates containing Mueller-Hinton agar using a swab. Subsequently, antimicrobial-impregnated discs were placed, and the plates were incubated (37 °C/18–24 h). The zones of inhibition were measured (mm), and the results obtained were recorded. The *S. aureus* ATCC 25923 strain was used for quality control.

The multiple antibiotic resistance (MAR) index was calculated for each isolate as the ratio (MAR = p/q) between the number of antimicrobials to which the isolate showed resistance (p) and the total number of antimicrobials tested (q) [21]. Considering it a source with frequent antibiotic exposure, an MAR greater than 0.20 was used [21,22].

### 2.7. Molecular Identification of Staphylococcus aureus and Detection of the mecA Gene

Genomic DNA was extracted from the bacterial suspension of the selected samples using the PureLink Genomic DNA Mini Kit (Invitrogen, Carlsbad, CA, USA), according to the manufacturer’s instructions. The extracted DNA was stored at −20 °C until the molecular analyses were performed.

Molecular identification of *Staphylococcus aureus* was performed by polymerase chain reaction (PCR) using primers Sa442-1 (5′-AAT CTT TGT CGG TAC ACG ATA TTC TTC ACG-3′; positions 5 to 34) and Sa442-2 (5′-CGT AAT GAG ATT TCA GTA GAT AAT ACA ACA-3′; positions 83 to 112). The reactions were performed in a thermal cycler, with adaptations of the parameters described by Martineau et al. [23], consisting of an initial denaturation at 93 °C for 3 min, followed by 40 cycles of denaturation at 95 °C for 1 s and annealing-extension at 55 °C for 30 s. The amplified products were subjected to electrophoresis on a 2% agarose gel stained with GelRed (Uniscience, Fremont, CA, USA) and visualized under ultraviolet light. Thus, the presence of a single band at 241 bp, when compared to the 100 bp molecular marker, was considered indicative of the presence of *Staphylococcus aureus*.

Detection of the *mecA* gene was also performed by PCR, but using the primers *mecA*1 (AAA ATC GAT GGT AAA GGT TGG) and *mecA*2 (AGT TCT GCA GTA CCG GAT TTG). The reactions were performed in a thermal cycler, using the adapted parameters described by Murakami et al. [24], with an initial denaturation at 94 °C for 5 min, followed by 40 cycles of denaturation at 94 °C for 30 s, primer annealing at 55.5 °C for 30 s, and extension at 72 °C for 1 min. The amplified products were analyzed by 2% agarose gel electrophoresis stained with GelRed (Uniscience, Fremont, CA, USA); the presence of a single band at 533 bp, when compared to a 100 bp molecular marker, was considered positive for the detection of the *mecA* gene.

Internationally recognized reference strains were used in all reactions performed: a positive control (*S. aureus* ATCC 33591) and a negative control (autoclaved ultrapure water).

### 2.8. Statistical Analysis

Statistical analyses were performed using R software (version 4.4.3). Initially, a descriptive analysis of the data was performed, determining the absolute and relative frequencies of antimicrobial resistance, along with the respective 95% confidence intervals (95% CI) using the Wilson method. The multiple antibiotic resistance (MAR) index was calculated for each isolate. The normality of MAR values was assessed using the Shapiro–Wilk test, and, as the assumptions of normality were met, the means of the mammal groups were compared using the *t*-test. To visualize the antimicrobial susceptibility profiles of the isolates, a heatmap was generated; the isolates were ordered in descending order based on the total number of resistances, and the antimicrobials were hierarchically clustered to identify similarity patterns [25]. Associations between the categorical variables, bacterial groups (CoPS and CoNS), multidrug resistance (MDR), molecular identification of *Staphylococcus aureus*, and animal groups (non-human primates and wild felids), were evaluated using Pearson’s chi-square test with Yates’ correction or Fisher’s exact test, as appropriate. Comparisons of antimicrobial resistance frequencies between non-human primates and wild felids were performed using Fisher’s exact test. To account for multiple comparisons across the ten antimicrobials evaluated, the Bonferroni correction was applied, resulting in an adjusted significance threshold of *p* < 0.005. These comparisons were considered exploratory due to the limited sample size. The significance level adopted was 5% (*p* < 0.05), except for the antimicrobial resistance comparisons, for which the Bonferroni-adjusted threshold was applied.

## 3. Results

It was possible to collect 37 samples: 22 from tufted capuchins (*Sapajus apella*) and 16 from wild felids, comprising one tiger (*Panthera tigris*), four pumas (*Puma concolor*), four southern tigrinas (*Leopardus guttulus*), three ocelots (*Leopardus pardalis*), one jaguar (*Panthera onca*), two jaguarundis (*Herpailurus yagouaroundi*), and a margay (*Leopardus wiedii*).

Of 37 mammal samples collected at the zoo, 83.7% (31/37) of the isolates were phenotypically identified as *Staphylococcus* spp. Of the total samples identified as *Staphylococcus* spp. from mammals, 35.5% (11/31) were isolated from felids and 64.5% (20/31) from non-human primates.

Furthermore, 61.3% (19/31) were identified as coagulase-negative staphylococci (CoNS), with 42.1% (8/19) isolated from felids and 57.9% (11/19) from non-human primates. While 38.7% (12/31) of the isolates were identified as coagulase-positive *Staphylococcus* (CoPS), 25% (3/12) were isolated from felids and 75% (9/12) from non-human primates. Despite the numerical difference, there was no statistically significant difference between the mammal groups (felids and non-human primates; *p* > 0.05; *p* = 0.4516; Fisher’s test) regarding the frequency of CoPS and/or CoNS isolates.

The resistance profile of the mammalian isolates is presented in Table 1. Regarding the prevalence of resistance, 56.2% of the feline isolates were resistant to antibiotics, whereas 65.5% of the non-human primate isolates were resistant to the antibiotics tested. There was no statistically significant difference in the overall resistance profile among the mammal groups (*p* > 0.05; *p* = 0.1165; Chi-square test with Yates’ correction).

Furthermore, Table 1 shows that 80.6% (25/31) of the isolates were classified as multidrug-resistant (MDR), that is, resistant to at least three or more classes of antimicrobials. Non-human primate isolates classified as MDR represent 80% (16/20) of the total samples in that group, a figure very similar to that observed in the felid group, where 81.8% (9/11) of the isolates are MDR (*p* > 0.05; *p* = 1; Fisher’s exact test).

The multiple antimicrobial resistance (MAR) index ranged from 0.1 to 1 (mean ± standard deviation: 0.58 ± 0.22). Consequently, the majority of bacterial isolates (90.3%; 28/31) exhibited a MAR index greater than 0.20. The highest MAR value (1) was isolated from a *Leopardus pardalis*, while the lowest (0.1) was isolated from a *Puma concolor*. The MAR was similar between the mammal groups (*p* > 0.05; *p* = 0.3; *t*-test), exceeding 0.2 for 90% (18/20) of non-human primate isolates (mean ± standard deviation of 0.52 ± 0.26) and 81.8% (9/11) of wild felid isolates (mean ± standard deviation of 0.62 ± 0.25; Table 1).

**Figure 1:**
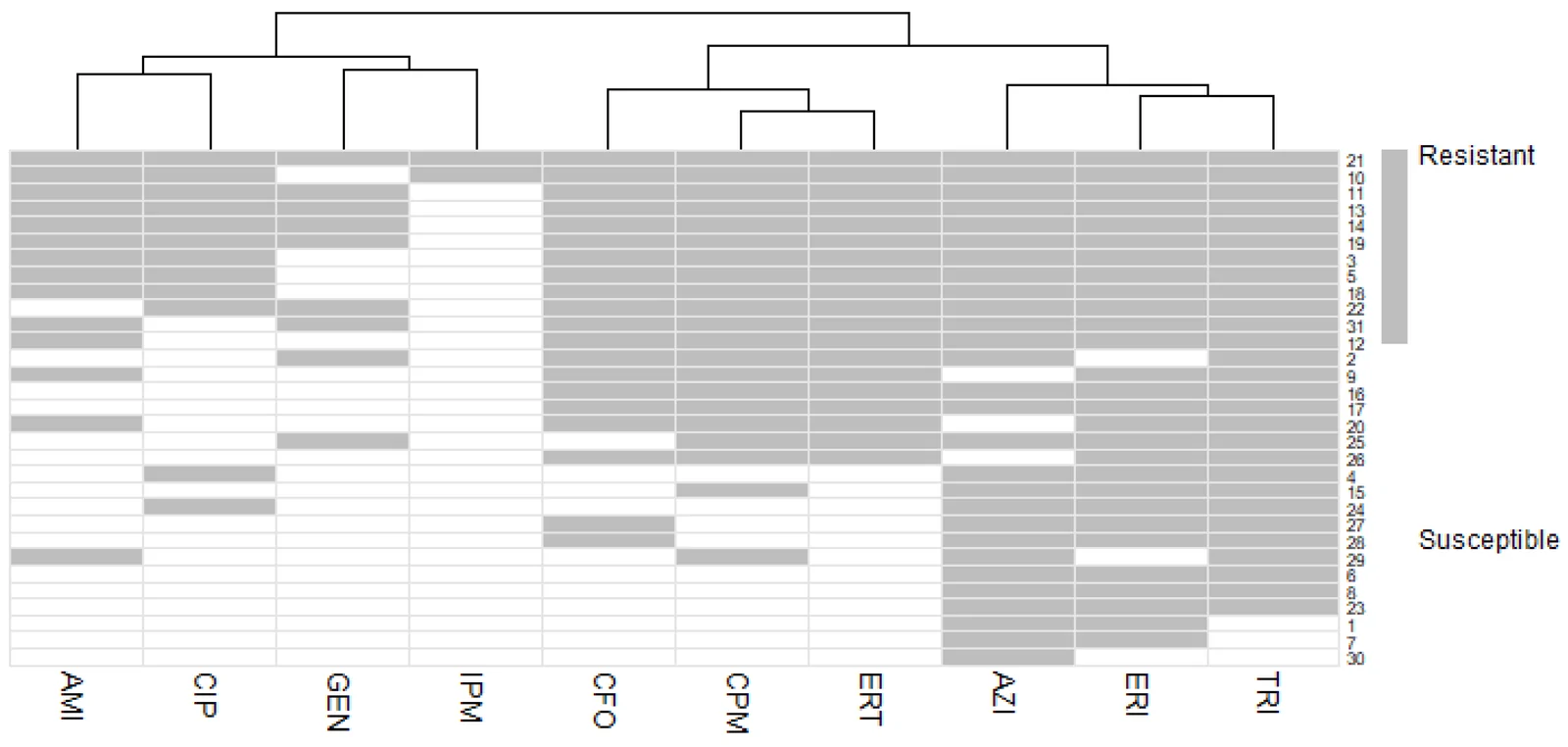
Heat map of antimicrobial susceptibility profiles of bacterial isolates. Legend: The rows represent the isolates, ordered from most resistant to most sensitive; gray cells indicate resistance, and white cells indicate susceptibility; AMI—Amikacin, AZI—Azithromycin, CIP—Ciprofloxacin, CFO—Cefoxitin, CPM—Cefepime, ERI—Erythromycin, ERT—Ertapenem, GEN—Gentamicin, IPM—Imipenem, TRI—Sulfamethoxazole-Trimethoprim. Source: Prepared by the authors.

**Figure 2:**
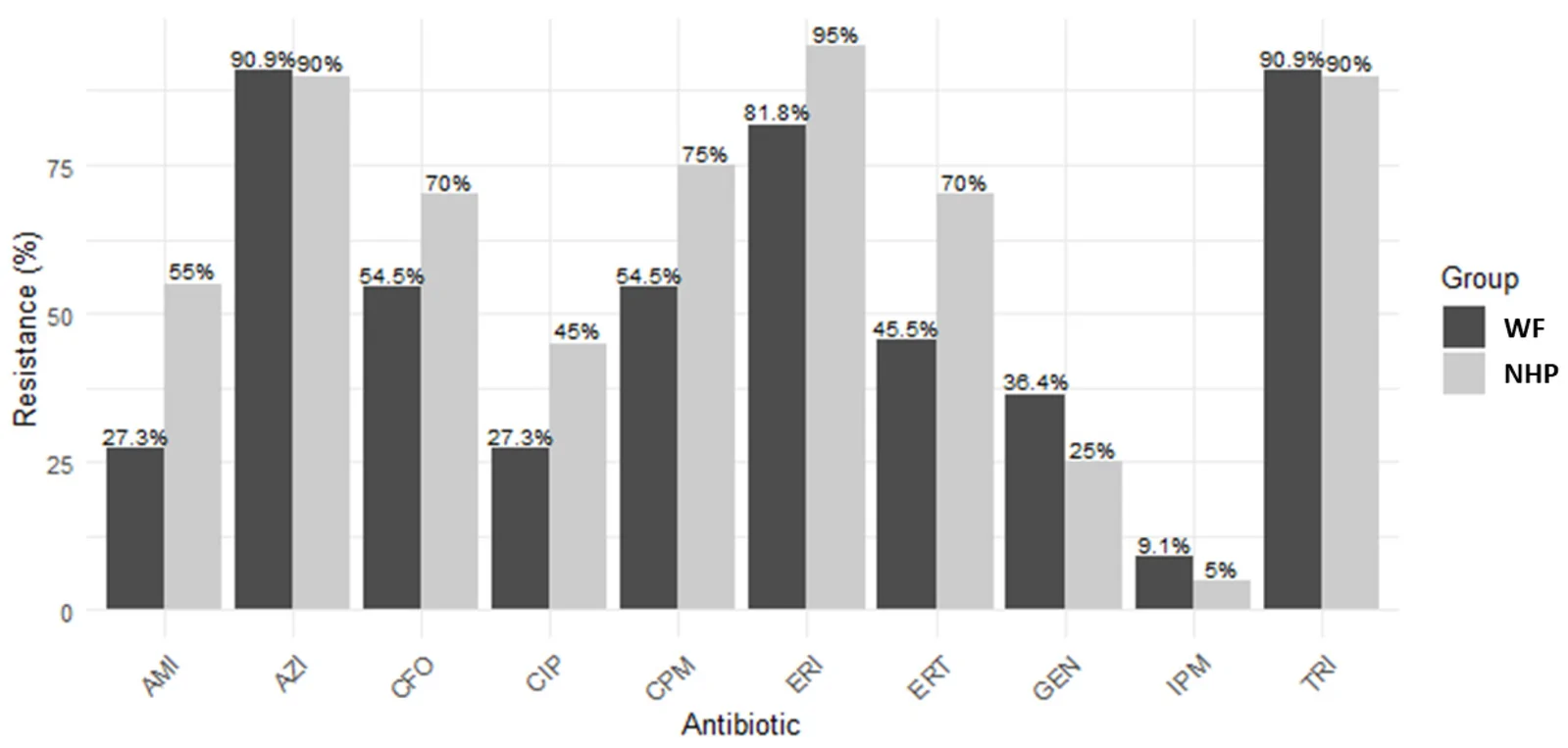
Resistance frequency of bacterial isolates to antibiotics and mammal group (wild felids and non-human primates). AMI—Amikacin (*p* = 0.258), AZI—Azithromycin (*p* = 1), CIP—Ciprofloxacin (*p* = 0.451), CFO—Cefoxitin (*p* = 0.452), CPM—Cefepime (*p* = 0.423), ERI—Erythromycin (*p* = 0.281), ERT—Ertapenem (*p* = 0.255), GEN—Gentamicin (*p* = 0.683), IPM—Imipenem (*p* = 1), TRI—Sulfamethoxazole-Trimethoprim (*p* = 1), WF—Wild Felines, NHP—Non-human primates. No statistically significant differences were detected between groups after Bonferroni correction (adjusted significance threshold: *p* < 0.005). Source: Prepared by the authors.

There was similarity between the resistance patterns of Azithromycin, Erythromycin and Sulfamethoxazole-Trimethoprim, all with 90.3% (28/31; 95%CI:75.1-96.7) of the isolates classified as resistant. Meanwhile, the most effective antibiotic for the isolates was imipenem, showing only 6.5% (2/31; 95% CI: 1.8–20.7) of resistant isolates (Figure 1). Although differences in antimicrobial resistance frequencies were observed between non-human primates and felids, none of the comparisons reached statistical significance after Bonferroni correction for multiple comparisons (adjusted *p*-values = 1.000) (Figure 2).

In addition to the phenotypic identification of isolates belonging to the genus *Staphylococcus*, *Staphylococcus aureus* was identified by PCR in 12.9% (4/31) of the isolates; these isolates originated from non-human primate samples (Table 1). The *mecA* gene was not detected in any of the isolates analyzed.

## 4. Discussion

Zoos are fundamental to wildlife conservation and the promotion of animal welfare, as they facilitate environmental education, conservation, and scientific research programs that help raise public awareness about the importance of biodiversity. Furthermore, the interaction among visitors, veterinarians, keepers, and animals makes this environment strategic for epidemiological surveillance, as it aids in detecting zoonotic risks and reinforces the understanding of One Health.

This study demonstrated a high prevalence of *Staphylococcus* spp. in the oral cavity of various wild mammals kept in captivity, including coagulase-positive isolates and *Staphylococcus aureus*. These data demonstrate that these animals can act as important reservoirs of the genus *Staphylococcus* in captive environments.

*Staphylococcus* spp. are opportunistic pathogens commonly found colonizing the skin and mucous membranes of humans and domestic animals [4,16]. However, the presence of pathogenic bacteria in captive animals is becoming increasingly frequent, linked to their proximity to humans and domestic animals; these animals can develop infectious diseases due to poor nutrition, lack of hygiene, management shortcomings, and pre-existing conditions [26,27].

Non-human primates (NHPs) play a fundamental role in ecosystem health, contributing to tropical forest regeneration through seed dispersal or acting as prey and predators within food webs [12,28]. However, due to the lack of food in these habitats, proximity to humans and domestic animals is becoming increasingly frequent. Furthermore, dietary changes can drive alterations in the oral microbiome of non-human primates, so much so that lower bacterial diversity is observed in captive individuals compared to those living in the wild [29,30]. *Staphylococcus* spp. are commonly found in the oral cavity of *Sapajus apella* [31]. In addition to this, similar pathogens have been found in the oral cavity of humans and NHPs [32]. These findings reinforce the potential of non-human primates (NHPs) as reservoirs and potential carriers of microorganisms resistant to various antimicrobials, given that they inhabit and move through diverse ecosystems, including forest, rural, and urban environments. This ecological interface facilitates the circulation and dissemination of resistant bacteria among wildlife, domestic animals, humans, and the environment, highlighting the role of these animals in the dynamics of antimicrobial resistance from a One Health perspective [22,23].

The high prevalence (83.7%) of isolates identified as *Staphylococcus* spp. in captive mammals, as demonstrated in this study, highlights the relevance of this pathogen to One Health; this finding aligns with those of De Alcântara et al. [7], who identified *Staphylococcus* spp. in 70.6% of samples from captive wild felids and canids, indicating that these microorganisms are part of the oral microbiota of wild felids. Other studies further show that large captive wild felids can also harbor antimicrobial-resistant *Staphylococcus* spp [13]. However, when the animal is immunosuppressed, the bacterial load tends to increase, resulting in an infection associated with the potential for antimicrobial resistance; if not adequately treated, this can pose a risk to the animal’s health [33]. Furthermore, captive animals typically have a history of stress due to complications preceding their life in the zoo; they also suffer from dietary changes or are immunocompromised, and these conditions can significantly influence the composition of the normal microbiota [34,35,36]. Animals with microbiomes in a state of dysbiosis associated with stress, intensive management, poor hygiene, or therapeutic interventions may be susceptible to infections by potentially resistant microorganisms [35,37]. Furthermore, the handling of captive animals by keepers may facilitate pathogen transmission through contact with feces or via accidental bites or scratches [38].

Another important factor to consider is the origin of the animals being evaluated. The mammals included in this study were rescued by environmental agencies following situations of vulnerability, such as wildlife trafficking, vehicle collisions, and other incidents requiring their removal from the natural environment. Typically, these animals arrive at zoos in a debilitated state due to the stressful conditions of intensive handling during capture, restraint, and transport, potentially exhibiting immunosuppression, nutritional imbalances, and a need for therapeutic interventions. These conditions can alter the microbiota, favoring dysbiosis and increasing the likelihood of colonization by pathogenic microorganisms that are potentially resistant to antimicrobials [34,35,36,37]. Thus, the high frequency of *Staphylococcus* spp. in the present study may stem not only from the characteristic microbiota of these animals but also from the clinical and environmental conditions to which they were subjected before and during their time in captivity.

In the present study, 38.7% of the isolates were identified as coagulase-positive staphylococci (CoPS), while only 12.9% were confirmed as *Staphylococcus aureus*, all of which were collected from non-human primates. The low frequency of *S. aureus* suggests that the species is not the primary colonizer of the oral cavity of the mammals evaluated. The predominance of coagulase-negative staphylococci (CoNS) observed in the present study is consistent with the findings of Sobreira et al. [30], who reported a higher prevalence of CoNS in wild non-human primates. A similar pattern is observed in domestic animals, such as swine [39,40]. CoNS are part of the normal microbiota of the skin and mucous membranes, playing a fundamental role in limiting colonization and invasion by opportunistic pathogens [41,42].

The majority of isolates from non-human primates (65.5%) and wild felids (56.2%) in this study exhibited resistance to the tested antibiotics, antibiotics commonly used in clinical practice to treat bacterial infections in both animals and humans. Although these groups of mammals differ in taxonomy and ecology, there was no significant difference between their antimicrobial resistance profiles. This suggests that these animals may be exposed to similar sources of resistant microorganisms while in captivity, possibly through contact with keepers, veterinarians, and the sharing of the same environment [6].

Furthermore, 80.6% of all isolates were resistant to three or more classes of antibiotics, being classified as multidrug-resistant (MDR) [8]. Meanwhile, 90.3% of the isolates exhibited a multiple antimicrobial resistance (MAR) index greater than 0.20, with a mean of 0.58, a value considered indicative of an origin in environments subject to high selective pressure from antimicrobials [21]. Similar results were reported by Sineke et al. [43], who found multidrug-resistant *Staphylococcus aureus* with a mean multiple-antibiotic resistance index of 0.50 in intensive pig farming in South Africa. Furthermore, all isolates identified as *Staphylococcus aureus* in the present study exhibited MAR values greater than 0.2. Both methods are used to characterize multiple antibiotic resistance profiles, and although the results are similar, the MAR method provides a quantitative measure of individual and group resistance, yielding more detailed results than the MDR method [44]. Thus, in the present study, the *Leopardus pardalis* isolate exhibited the highest MAR value, suggesting greater exposure to sources of selection for antibiotic-resistant bacteria, whereas the *Puma concolor* isolate showed the lowest index. Although both isolates originated from felids, there was no significant difference in mean MAR values between non-human primates and wild felids, indicating that the groups may be subject to the same level of exposure to resistant *Staphylococcus* spp. in the captive environment. Similar mean MAR values were found in a study involving swine [43].

The major issue regarding the presence of resistant *Staphylococcus* spp. in captive wild animals is antimicrobial resistance, which increases mortality rates due to the difficulty of providing adequate treatment; this necessitates meticulous oversight and continuous health monitoring of captive animals given the risk of transmission. Saad, Salama, and Ibrahim [13] also observed high antibiotic resistance in *Staphylococcus* spp. isolated from fecal samples of captive African lions from Egypt’s national circus, revealing the potential zoonotic risk posed by these bacteria isolated from lions.

The majority of the antibiotics tested, such as azithromycin, erythromycin, sulfamethoxazole-trimethoprim, cefepime, cefoxitin, and ertapenem, proved ineffective against *Staphylococcus* spp. isolates. Meanwhile, the antibiotic that demonstrated the greatest efficacy against the bacterial isolates was Imipenem, with 93.3% of the isolates being susceptible. These data corroborate the findings of Sobreira et al. [30], who identified erythromycin resistance in *Staphylococcus* spp. isolates from the oral cavity of *Sapajus libidinosus* capuchin monkeys in northeastern Brazil. Furthermore, studies by Saad, Salama, and Ibrahim [13] also demonstrated resistance to cefepime and cefoxitin in *Staphylococcus* spp. isolated from fecal samples of captive African lions from the Egyptian National Circus.

The differences in antimicrobial resistance observed across various studies may be related to the diverse origins of the animals, which involve distinct management practices, the genetic diversity of bacterial populations, and, above all, the selective pressure exerted by antimicrobial use [45,46].

The prevalence of methicillin-resistant *Staphylococcus aureus* (MRSA) isolates has been reported as low in wild animals, such as non-human primates and felids, although their detection and investigation are of sanitary significance [27,47]. In the present study, there was no molecular evidence of resistance related to the presence of the *mecA* gene, but other mechanisms associated with resistance in *Staphylococcus* spp. were not investigated. Indeed, isolate resistance may also have been mediated by alternative mechanisms, such as homologous genes like *mecC*; for instance, research by Loncaric et al. [48] demonstrated that oxacillin-resistant *Staphylococcus* spp. isolates from wild animals did not test positive for the *mecA* gene via conventional PCR, yet all tested positive for the *mecC* gene via specific PCR. Other factors, such as the overproduction of β-lactamases, modifications to penicillin-binding proteins, and even biofilm formation, can significantly influence and increase antimicrobial resistance [49,50,51]. Thus, the absence of the *mecA* gene does not rule out the important epidemiological role of these isolates.

Despite not being widely exposed to antimicrobials like other animal groups, wild mammals kept in captivity have been shown to act as potential reservoirs for *Staphylococcus* spp., including methicillin-resistant *Staphylococcus aureus* (MRSA) [27]. Thus, the increased presence of these bacteria in the oral cavities of these animals may be linked not only to the therapeutic use of antimicrobials in captive animals but, more importantly, to frequent contact with humans, such as keepers, veterinarians, and handling practices, as well as to diet, water, or even environmental contamination. This situation is further aggravated by the risk of accidents involving animal handlers through contact with excrement or via bites or scratches, as demonstrated by Devaux et al. [38], who described the transmission of *Staphylococcus* spp. to humans via non-human primate bites.

Studies demonstrate that changes in the environment and diet of wild animals can influence the composition of the gut microbiota, comparing free-ranging animals with those in captivity, and that the latter may face a higher risk of developing infectious diseases [52,53]. Thus, antimicrobial resistance is a serious One Health issue; however, the situation is further exacerbated when it comes to antibiotic resistance in wild and captive animals, due to the lack of monitoring and information [10,11,54].

Infections caused by resistant microorganisms are difficult to resolve and often increase morbidity and mortality rates in humans and animals. Thus, the findings reinforce the need for periodic monitoring of antimicrobial resistance and measures to control the spread of resistant *Staphylococcus* in captive environments, in order to protect animals, keepers, veterinarians, and zoo visitors.

### Study Limitations

Although this study provides important information on antimicrobial resistance in *Staphylococcus* spp. isolates from captive wild mammals, some limitations should be considered. The study was conducted at a single zoo, with a relatively small number of isolates and unequal representation of animal species. The small sample size may have limited the statistical power to detect differences in antimicrobial resistance between groups. The grouping of different wild felid species may have introduced heterogeneity, and the absence of statistically significant differences should not be interpreted as evidence of equivalence between groups. Furthermore, the cross-sectional design, the absence of environmental and human samples, and the lack of systematic data on prior antimicrobial exposure in this study make it difficult to determine possible transmission events. Molecular identification at the species level was limited, and molecular assessment was restricted to the presence of the *mecA* gene. Therefore, future research should include a larger number of zoos and bacterial isolates, despite the limitations associated with the restraint and handling of wild animals. In addition to investigating other mechanisms that determine resistance (such as *mecC*), virulence factors and biofilm formation, future research should explore potential alternatives for the prevention and treatment of staphylococcal infections in captive animals. These new analyses could aid in a better understanding of the epidemiology of these pathogens, in addition to contributing to strategies for the prevention and control of microbial resistance within the One Health context.

## 5. Conclusions

This study demonstrated a high prevalence of *Staphylococcus* spp. (83.7%) in the oral cavity of captive wild mammals, with a predominance of coagulase-negative staphylococci and the identification of *Staphylococcus aureus* in 12.9% of the isolates, exclusively from non-human primates. Furthermore, more than half of the isolates (56.2% from wild felids and 65.5% from non-human primates) showed resistance to the tested antimicrobials; 80.6% were classified as multidrug-resistant, and 90.3% exhibited a multiple antimicrobial resistance index indicative of an origin in environments under high selective pressure from antimicrobials, demonstrating the widespread circulation of bacteria across different organisms and environments. These findings highlight the potential of captive wild animals as reservoirs of *Staphylococcus* spp. multidrug-resistant to conventional antimicrobials, suggesting potential transmission across the human–animal-environment interface. In this context, this One Health interface requires continuous epidemiological surveillance and biosecurity measures, given that interactions among animals, caretakers, veterinarians, and zoo visitors can facilitate exposure to resistant microorganisms and the risk of zoonotic transmission, with implications for animal production and public health.

## Figures and Tables

**Table 1:** Bacterial resistance profile of isolates from samples collected from the oral cavity of mammals (non-human primates and wild felids) at a zoo in southern Brazil.

Sample			Antibiotics												Identification	
			Aminoglycoside	Macrolide	Fluoroquinolone	Cephalosporin	Cephalosporin	Macrolide	Carbapenem	Aminoglycoside	Carbapenem	Sulfonamides	MDR	MAR	Phenotypic	Genotypic
		Animal	AMI	AZI	CIP	CFO	CPM	ERI	ERT	GEN	IPM	TRI			ID	*Sa*
1	1 PM	*Sapajus apella*	S	R	S	S	S	R	S	S	S	S	0	0.2	CoPS	A
2	2 PM	*Sapajus apella*	S	R	S	R	R	S	R	R	S	R	1	0.6	CoPS	P
3	3 PM	*Sapajus apella*	R	R	R	R	R	R	R	S	S	R	1	0.8	CoPS	P
4	4 PM	*Sapajus apella*	S	R	R	S	S	R	S	S	S	R	1	0.4	CoNS	A
5	5 PM	*Sapajus apella*	R	R	R	R	R	R	R	S	S	R	1	0.8	CoPS	P
6	6 PM	*Sapajus apella*	S	R	S	S	S	R	S	S	S	R	0	0.3	CoPS	P
7	7 PM	*Sapajus apella*	S	R	S	S	S	R	S	S	S	S	0	0.2	CoPS	A
8	8 PM	*Sapajus apella*	S	R	S	S	S	R	S	S	S	R	0	0.3	CoPS	A
9	9 PM	*Sapajus apella*	R	S	S	R	R	R	R	S	S	R	1	0.6	CoPS	A
10	10 PM	*Sapajus apella*	R	R	R	R	R	R	R	S	R	R	1	0.9	CoPS	A
11	12 P	*Sapajus apella*	R	R	R	R	R	R	R	R	S	R	1	0.9	CoNS	A
12	13 P	*Sapajus apella*	R	R	S	R	R	R	R	S	S	R	1	0.7	CoNS	A
13	15 P	*Sapajus apella*	R	R	R	R	R	R	R	R	S	R	1	0.9	CoNS	A
14	16 P	*Sapajus apella*	R	R	R	R	R	R	R	R	S	R	1	0.9	CoNS	A
15	17 P	*Sapajus apella*	S	R	S	S	R	R	S	S	S	R	1	0.4	CoNS	A
16	18 P	*Sapajus apella*	S	R	S	R	R	R	R	S	S	R	1	0.6	CoNS	A
17	19 P	*Sapajus apella*	S	R	S	R	R	R	R	S	S	R	1	0.6	CoNS	A
18	20 P	*Sapajus apella*	R	R	R	R	R	R	R	S	S	R	1	0.8	CoNS	A
19	21 P	*Sapajus apella*	R	R	R	R	R	R	R	R	S	R	1	0.9	CoNS	A
20	22 P	*Sapajus apella*	R	S	S	R	R	R	R	S	S	R	1	0.6	CoNS	A
21	01 J	*Leopardus pardalis*	R	R	R	R	R	R	R	R	R	R	1	1	CoNS	A
22	02 J	*Leopardus pardalis*	S	R	R	R	R	R	R	R	S	R	1	0.8	CoNS	A
23	01 GM	*Leopardus guttulus*	S	R	S	S	S	R	S	S	S	R	0	0.3	CoPS	A
24	02 GM	*Leopardus guttulus*	S	R	R	S	S	R	S	S	S	R	1	0.4	CoNS	A
25	01GMOU	*Herpailurus yagouaroundi*	S	R	S	S	R	R	R	R	S	R	1	0.6	CoNS	A
26	02 GMOU	*Herpailurus yagouaroundi*	S	S	S	R	R	R	R	S	S	R	1	0.5	CoNS	A
27	03 GM	*Leopardus guttulus*	S	R	S	R	S	R	S	S	S	R	1	0.4	CoPS	A
28	01 MARA	*Leopardus wiedii*	S	R	S	R	S	R	S	S	S	R	1	0.4	CoPS	A
29	01 T	*Panthera tigris*	R	R	S	S	R	S	S	S	S	R	1	0.4	CoNS	A
30	3 PM	*Puma concolor*	S	R	S	S	S	S	S	S	S	S	0	0.1	CoNS	A
31	01 OP	*Panthera onca*	R	R	S	R	R	R	R	R	S	R	1	0.8	CoNS	A

Legend: R—Resistant, S—Susceptible, MDR—Multidrug-resistant, 0—No (Multidrug-resistant), 1—Yes (Multidrug-resistant), MAR—Multiple Antibiotic Resistance Index, *Sa*—*Staphylococcus aureus*, P—Present, A—Absent, AMI—Amikacin, AZI—Azithromycin, CIP—Ciprofloxacin, CFO—Cefoxitin, CPM—Cefepime, ERI—Erythromycin, ERT—Ertapenem, GEN—Gentamicin, IPM—Imipenem, TRI—Sulfamethoxazole-Trimethoprim. Source: Prepared by the authors.

## Data Availability

The original contributions presented in this study are included in the article. Further inquiries can be directed to the corresponding author.
